# Impact of sensor configuration and melanin concentration on reflective pulse oximetry using Monte Carlo simulations

**DOI:** 10.1038/s41598-025-26560-6

**Published:** 2025-11-10

**Authors:** Maximilian Reiser, Andreas Breidenassel, Oliver Amft

**Affiliations:** 1 Faculty of Electrical Engineering Landshut, HAW Landshut, Landshut, Germany; 2 Intelligent Embedded Systems Lab., University of Freiburg, Freiburg, Germany; 3 Hahn-Schickard, Freiburg, Germany

**Keywords:** Biomedical engineering, Optical sensors

## Abstract

We investigate the impact of melanin concentration C_Mel_ and photoplethysmography (PPG) sensor configuration on signal quality and estimation accuracy of oxygen saturation $\mathrm {SpO_2}$. We deploy Monte Carlo (MC) simulations of photon-skin interactions to estimate arterial oxygen saturations $\mathrm {SaO_2}$ ranging from 70 to 100% and melanin concentration C_Mel_ ranging from 2.55 to 30.5%. We analysed the effects of red and infrared wavelengths (624 nm, 660 nm, 850 nm, and 940 nm), beam profiles (LED and VCSEL), as well as beam incidence angles of light sources (0°, 45°, and − 45°) on Perfusion Index (PI), reflective $\mathrm {SpO_2}$ estimation, and signal-to-noise ratio (SNR) for source-detector distances ranging from 2  mm to 9 mm. Maximum PI was observed for in the red spectral range at 624 nm and in the infrared spectral range at 940 nm. In contrast, reflective pulse oximetry provided more accurate results at 660 nm and 850 nm, independent of wavelength combination. We conclude that the VCSEL beam profile at 0° is the optimal light source for a wide range of applications, as it offers a balance between PI and absolute $\mathrm {SpO_2}$ estimations error.

## Introduction

The photoplethysmography (PPG) curve is an optical method to measure the change in blood volume due to heartbeat. The PPG curve can be used to determine a variety of vital parameters (e.g., heart rate, arterial stiffness, oxygen saturation) ^1–3^. Light, typically in the green, red, or infrared spectral range, is sent into tissue and the unabsorbed fraction of light is measured on the skin surface using a detector (e.g., photodiode). Fluctuations in the detected light intensity *I*, as a result of systole and diastole, represent the pulsatile change in blood flow. The PPG curve can be measured transmissive (through thin body parts) or reflective (on the same skin surface). In particular, reflective measurements are suitable for wearable devices, including smartwatches, and can have a clinical benefit, e.g., early diagnosis, monitoring, or preventing further health complications ^3^. The perfusion index (PI) is derived from the PPG curve and used to describe the ratio of pulsating to non-pulsating components of the detected light intensity *I*. Non-pulsating components represent the static part (DC level) of the PPG curve, which is primarily caused by baseline blood volume, skin, muscles, connective tissue, or bones. The pulsating components of the detected light intensity *I* represent the dynamic part (AC level) of the PPG curve, which is caused by the pulsatile blood flow.

Pulse oximetry uses two light sources with different wavelengths, typically in the red and infrared spectral range, to estimate the peripheral oxygen saturation $\mathrm {SpO_2}$. $\mathrm {SpO_2}$ is an estimate of arterial oxygen saturation $\mathrm {SaO_2}$, which is usually measured directly by blood gas analysis. Oxygenated hemoglobin $\textrm{O}_{2}\textrm{Hb}$ and deoxygenated hemoglobin $\textrm{HHb}$ have different absorption characteristics in the red and infrared spectral range. The ratio of ratios (RoR) can be calculated based on the ratio of the PI of the red wavelength to the PI of the infrared wavelength ^4^. RoR is used in manufacturer-specific calibration curves or equations to estimate $\textrm{SpO}_{2}$. Estimated $\mathrm {SpO_2}$ is an indirect measurement and therefore only an approximation, but it has been shown to be consistent with actual arterial oxygen saturation $\mathrm {SaO_2}$ in clinical practice ^5^. Pulse oximetry is non-invasive, can continuously estimate $\mathrm {SpO_2}$, and is suitable for clinical and home applications, due to its user-friendliness. Estimated $\mathrm {SpO_2}$ is a key vital parameter that provides information on a patient’s respiratory system status and is essential in the diagnosis and treatment of numerous diseases (e.g., COVID) ^5^. The reliability of $\mathrm {SpO_2}$ estimation is influenced by various factors, including skin pigmentation ^5–7^.

Melanin is a pigment that is responsible for the skin type and influences $\mathrm {SpO_2}$ estimation due to its high absorption coefficient $\mu _a$ ^8^. The increased photon absorption leads to a reduction in detected photons and can result in an incorrect $\mathrm {SpO_2}$ estimation. Melanin concentration $C_\textrm{Mel}$ affects $\mathrm {SpO_2}$ estimation accuracy, and hence is critical for patient health monitoring ^6,9^. The deviation in $\mathrm {SpO_2}$ estimation of today’s pulse oximeters increases with increasing melanin concentration $C_\textrm{Mel}$ ^9^. In particular, with decreasing arterial oxygen saturation $\mathrm {SaO_2}$, it was found that for individuals with darker skin pigmentation significant deviations between $\mathrm {SpO_2}$ estimation and actual arterial oxygen saturation $\mathrm {SaO_2}$ occurred ^6,9^. In critical treatment scenarios, including low oxygen saturation, any inaccuracy in $\mathrm {SpO_2}$ estimation may result in incorrect treatment decisions.

Typically, a light-emitting diode (LED) is used as light source for PPG curve measurements and $\mathrm {SpO_2}$ estimation. As surface emitters, LEDs show a Lambertian emission characteristic with deviations due to chip technology or additional optical elements. Vertical cavity surface emitting lasers (VCSELs) emit light perpendicular to the surface and have a low beam divergence (typically between 18° and 25°). Thus VCSELs may be suitable for wearables and applications that require control over the beam incidence angle. The trajectories of detected photons in reflective PPG measurements are shaped according to a banana curve ^10^. The banana curve of the detected photons is influenced by the beam incidence angle of photons entering skin tissue ^11^. Photons with a positive incidence angle directed towards the detector yield a more “flat” banana curve, i.e. the curve is closer to the skin surface, than if photons enter tissue perpendicularly, i.e. at 0°. Photons with an incidence angle directed away from the detector, i.e. <0°, travel at a relatively longer photon path compared to those entering with angles $\ge$0°, and thus penetrate deeper into tissue.

For $\mathrm {SpO_2}$ estimation, wavelengths in the red spectrum between 620 nm and 660 nm and in the infrared spectrum between 850 nm and 940 nm are typically used. The optical characteristics of the skin vary depending on the wavelength, as each wavelength interacts differently with tissue chromophores (e.g., melanin and hemoglobin). Small variations in the selection of wavelengths within the red and infrared spectrum directly affect PI ^12^. Large PI is often considered as an indicator of good signal quality, yet is unclear whether an increasing PI is linked to a more precise $\mathrm {SpO_2}$ estimation.

Monte Carlo (MC) simulations are used to analyse photon-tissue interactions ^12–15^. With MC methods, the behaviour of photons that traverse tissue are modelled to extract detailed insights of scattering, absorption, and photon paths. MC simulations are an established tool in biomedical optics to analyse complex tissue properties ^15–17^. MC simulations can provide insight into how photons interact depending on arterial oxygen saturation $\mathrm {SaO_2}$ and melanin concentration $C_\textrm{Mel}$. In addition, MC simulations could provide a detailed understanding of how photons of specific wavelengths interact with $\textrm{O}_{2}\textrm{Hb}$ and $\textrm{HHb}$, which is fundamental for $\mathrm {SpO_2}$ estimation.

Earlier work has analysed photon pathlength, penetration depth, RoR, and the relative contributions of skin sublayers in both reflectance and transmittance mode PPG in the red and infrared wavelength spectrum ^18–20^. Several investigations based on MC simulations have analysed sources of $\mathrm {SpO_2}$ estimation error. Early analyses varied depth and magnitude of arterial pulsation to explore calibration sensitivity, while more recent studies explicitly model the impact of skin pigmentation (melanin concentration) on AC level, calibration curves, and measurement error ^14,21–23^. However, prior MC simulations covered a limited subset of wavelengths (e.g., 660 nm and 940 nm), simplified source geometries (e.g., normal incidence), or focused on a single performance metrics ^13,14,20,22,24,25^. Few studies have jointly analysed how wavelength selection and source-detector geometry interact with melanin concentration to shape PI, signal-to-noise ratio (SNR), and $\mathrm {SpO_2}$ estimation error across relevant parameter ranges.

In this work, we present a comprehensive MC simulation analysis that systematically varies red and infrared wavelengths (624 nm, 660 nm, 850 nm, and 940 nm), light source beam profiles (LED and VCSEL), and beam incidence angles of VCSEL (0°, 45°, − 45°) across source–detector distances (2 to 9 mm). We take into account different melanin concentrations $C_\textrm{Mel}$ ranging from 2.55 to 30.5% and arterial oxygen saturations $\mathrm {SaO_2}$ ranging from 70 to 100%. Since spectral bandwidth effects of light sources have been previously examined ^26^, we focus on source-detector geometry, including beam profile, incidence angle, and source-detector distance). By quantifying PI, SNR, and $\mathrm {SpO_2}$ estimation error within a unified framework, we identify pulse oximeter configurations that simultaneously mitigate pigmentation-related error and preserve signal quality. Our main contributions are as follows:

1. We show that wavelength variations in red and infrared spectral ranges have contrary effects on PI and $\mathrm {SpO_2}$ estimation, which has not been reported to date.
2. We compare beam profiles and beam incidence angles of VCSEL and conventional LEDs for varying source-detector distances ranging from 2 to 9 mm.
3. We provide approaches for more accurate and reliable $\mathrm {SpO_2}$ estimation in pulse oximeters, regardless of individual skin pigmentation, thus contributing to more equal medical care.

## Methods

**Fig. 1:**
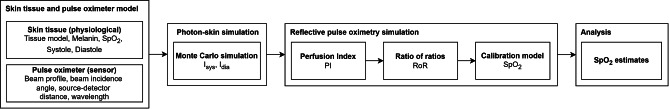
Schematic overview of the simulation pipeline. The workflow consists of four stages: (1) skin tissue and pulse oximeter modelling (skin physiology and sensor configuration), (2) photon–skin simulation using Monte Carlo methods, (3) reflective pulse oximetry simulation including perfusion index (PI), ratio of ratios (RoR), and calibration of $\mathrm {SpO_2}$ estimation, and (4) analysis using mean absolute error (MAE) and signal-to-noise ratio (SNR).

Our approach comprises three key components: (1) anatomy modelling of skin tissue and the PPG sensor system, (2) photon-skin MC simulation, (3) reflective pulse oximeter simulation (see Fig. 1). The skin tissue model is based on established characteristics and parameters. The pulse oximeter model includes a generalised population calibration model that covers all photon-skin simulations. Furthermore, the pulse oximeter sensor model includes configuration options for emitted light wavelength, beam profile, beam incidence angle, and source-detector distance. Our evaluation includes varying skin types (melanin concentration C_Mel_) and arterial oxygen saturation $\mathrm {SaO_2}$ values.

### Skin and sensor modelling

An anatomical skin tissue model was developed as a semi-infinite layered model. Skin was represented in six layers according to Moço et al.^12^: *epidermis*, $capillary\ loops$, $upper\ plexus$, $reticular\ dermis$, $deep\ plexus$, and *hypodermis*. The thickness of *epidermis* was reduced ^27^ and a layer *muscle* was added ^14^. Skin layer characteristics are detailed in Table 1. Absorption coefficients $\mu _a$ and scattering coefficients $\mu _s$ of the six skin layers were calculated according to Moço et al. ^12^. The optical properties of $\textrm{O}_{2}\textrm{Hb}$ and $\textrm{HHb}$ were derived from Bosschaart et al. ^28^ and optical parameters of water were taken from Hale and Querry ^29^. The absorption coefficients $\mu _a$ and scattering coefficients $\mu _s$ for *muscle* and *hypodermis* were adapted from Simpson et al. ^30^, and the absorption properties of melanin were based on Kim et al. ^31^. In the present study, we simulated the physiological states of systole and diastole to determine PI. The fractional volume of arterial blood was increased to model systole of the skin layers (see Table 1), following Moço et al. ^12^.

**Table 1:** Skin characteristics (refractive index *n*, layer thickness $d_t$, blood concentration $C_b$, water concentration $C_w$, and vessel diameter $v_d$) for layers *epidermis*, *capillary* *loops*, *upper* *plexus*, *reticular* *dermis*, *deep* *plexus*, and *hypodermis* ^12,27^.

Skin layer	*n*	$d_t$ [mm]	$C_b$	$C_w$	$v_d$ [mm]	Blood oscillation
Epidermis	1.33	0.27	0	0.20	0	No
Capillary loops	1.37	0.15	0.004	0.65	0.01	Yes
Upper plexus	1.40	0.08	0.02	0.65	0.02	Yes
Reticular dermis	1.40	1.2	0.004	0.65	0.02	Yes
Deep plexus	1.40	0.50	0.04	0.65	0.04	Yes
Hypodermis	1.44	0.55	0.03	0.65	0.05	Yes

**Fig. 2:**
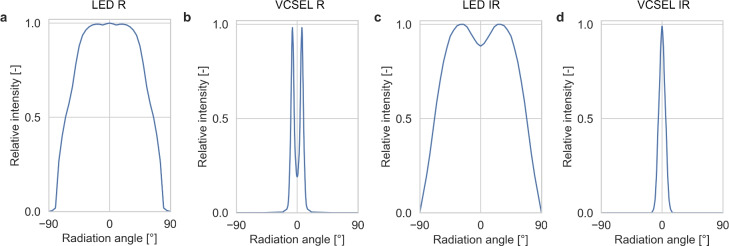
Radiation characteristics of light sources for spatial distribution in air and wavelength combinations of the red (624 nm, 660 nm) and infrared (850 nm, 940 nm) spectrum used in our analyses. (**a**) $\textrm{LED}_\textrm{R}$. (**b**) $\textrm{VCSEL}_\textrm{R}$. (**c**) $\textrm{LED}_\textrm{IR}$. (**d**) $\textrm{VCSEL}_\textrm{IR}$.

We implemented four different sensor configurations based on real light sources and a detector with a sensitivity curve depending on wavelength and beam incidence angle. Beam profiles and detectors sensitivity curves represent typical shapes for the respective devices. For the red spectrum, we implemented the beam profile $\textrm{LED}_\textrm{R}$ based on LED KR EGLP41.22 and $\textrm{VCSEL}_\textrm{R}$ based on VCSEL V100P000A-680, both from OSRAM Opto Semiconductors GmbH. For the infrared spectrum, we implemented the beam profile $\textrm{LED}_\textrm{IR}$ based on LED SFH 4043 and $\textrm{VCSEL}_\textrm{IR}$ based on VCSEL PLPVYL1 940A_E, both from OSRAM Opto Semiconductors GmbH. Figure 2 shows the relative intensity over the radiation angle of the beam profiles implemented from the light sources. For the detector, we implemented the photodiode SFH 2704 from OSRAM Opto Semiconductors GmbH. Source-detector distances ranged from 2 mm to 9 mm.

The beam incidence angles of 0° (perpendicular to the skin’s surface), 45° (towards the direction of the detector), and -45° (in opposite direction of the detector) were implemented for the beam profiles of the light source VCSEL ($\textrm{VCSEL}_\textrm{R}$ and $\textrm{VCSEL}_\textrm{IR}$). The beam profile of LEDs ($\textrm{LED}_\textrm{R}$ and $\textrm{LED}_\textrm{IR}$) were simulated with perpendicular emission onto the skin surface (beam incidence angle of 0°). For each combination of beam profile and beam incidence angle, the wavelengths 624 nm, 660 nm, 850 nm, and 940 nm were simulated.

### Photon-skin simulation

The photon-skin simulation was based on a validated MC simulation framework for photon-tissue interactions ^32^. The initial emission direction was sampled from the beam profile (see Fig. 2) using inverse-transform sampling. The intensity $I(\theta )$ was normalised to a probability density $p(\theta )$, and the cumulative distribution $F(\theta )=\int _0^\theta p(\theta ') \mathrm d\theta '$ was tabulated as a lookup table. For each photon packet, a random number $u \sim \mathscr {U}(0,1)$ was generated and the corresponding emission angle was obtained as $\theta = F^{-1}(u)$ by linear interpolation between neighbouring entries of the lookup table. For rotationally symmetric profiles, the azimuthal angle was chosen uniformly as $\phi \sim \mathscr {U}(0,2\pi )$.

At tissue entry, a fraction of photons in a photon packet was reflected according to the Fresnel equations for unpolarised light with:

1 $$\begin{aligned} R_{spec}=\tfrac{1}{2}(R_s+R_p), \end{aligned}$$

where $R_s$ and $R_p$ denote Fresnel reflectance coefficients for s- (perpendicular) and p- (parallel) polarized light, respectively. Photon packet weight *w* was updated accordingly ^15^:

2 $$\begin{aligned} w = 1 - R_{spec}. \end{aligned}$$

After each scattering event, the photon pathlength *l* was determined as:

3 $$\begin{aligned} l = - \frac{\ln (\xi )}{\mu _s}, \end{aligned}$$

where $\xi$ is a uniformly distributed random number in [0, 1] and $\mu _s$ is the scattering coefficient. At tissue boundaries, $\Delta l$ was updated and the photon packet was either transmitted or reflected based on the reflectance R ^15^:

4 $$\begin{aligned} R= {\left\{ \begin{array}{ll} \frac{(n_i-n_t)^2}{(n_i+n_t)^2} & \text {if } \theta _i = 0,\\ \frac{1}{2}[\frac{sin^2(\theta _i-\theta _t)}{sin^2(\theta _i+\theta _t)}+\frac{tan^2(\theta _i-\theta _t)}{tan^2(\theta _i+\theta _t)}] & \text {if } 0< \theta _i< \theta _c, \\ 1 & \text {if } \theta _c< \theta _i < \frac{\pi }{2}, \end{array}\right. } \end{aligned}$$

where $\theta$ is the photon packet angle (*i*: incidence angle, *t*: transmission angle), *n* the refractive index (*i*: incidence tissue, *t*: transmission tissue). If R was smaller than a randomly generated number $\xi$, the photon packet was transmitted, otherwise reflected. At each scattering event, the new scattering angle $\theta$ was sampled from the Henyey-Greenstein phase function ^33^ with the anisotropy factor *g*:

5 $$\begin{aligned} p(\theta )=\frac{1}{4\pi }\frac{1-g^2}{(1+g^2-2g\ cos\theta )^\frac{3}{2}} \end{aligned}$$

and the azimuth angle $\phi$ was chosen randomly from $[0,2\pi ]$. The photon packet weight was updated to account for absorption ^15^ as:

6 $$\begin{aligned} w = w - \Big (w\times \frac{\mu _a}{\mu _s+\mu _a}\Big ). \end{aligned}$$

Photon packets that left tissue and hit the detector were recorded according to the photodiode sensitivity curve. Normalised light intensity *I* was calculated as follows ^34^:

7 $$\begin{aligned} I^* = \frac{1}{N_d}\sum _{i=1}^{N_d} w_{d_i}, \end{aligned}$$

where $I^*$ is derived as $I_{\textrm{sys}}$ and $I_{\textrm{dia}}$, $N_d$ is the number of detected photon packets and $w_d$ the weight of each detected photon packet.

The MC simulation framework has been validated in prior works against laboratory measurements of a physical porcine skin phantom and a participant study with a wearable prototype ^11,32,35^. We showed that measurements are in close agreement with the MC simulations regarding the non-pulsatile reflective PPG DC level, its variation with wavelength, and its dependence on source–detector geometry (beam incidence angle and source–detector distance). Based on the above results, we confirmed that the MC simulation captures DC-level trends relevant for reflective PPG design. Our validation results demonstrate that the MC simulation framework can describe photon transport in skin, geometry-dependent light collection, and provides a solid basis for the pulsatile simulations performed in the present study. Furthermore, we validated real-world PPG measurements (systolic and diastolic signal levels) from a participant study against our MC simulation framework, including beam profiles. The measured and simulated results were in close agreement, confirming that the framework accurately reproduces real-world photon-skin interactions.

### Reflective pulse oximetry simulation

Pulse oximeters use the PI values from the red and infrared spectral range to estimate $\mathrm {SpO_2}$. PI was calculated as the ratio of the dynamic change in blood volume, caused by systole and diastole, to the static component of the reflected light:

8 $$\begin{aligned} \textrm{PI} = \frac{I_{\textrm{dia}} - I_\textrm{sys}}{I_\mathrm {{sys}}}. \end{aligned}$$

Using the PI values from wavelengths with opposing relative absorption characteristics for $\textrm{O}_{2}\textrm{Hb}$ and $\textrm{HHb}$, RoR can be calculated:

9 $$\begin{aligned} \textrm{RoR} = \frac{\textrm{PI}_{\lambda _{\textrm{R}}}}{\textrm{PI}_{\lambda _{\textrm{IR}}}}, \end{aligned}$$

where $PI_{\lambda _\textrm{R}}$ is the PI of a wavelength in the red spectral range (624 nm or 660 nm) and $PI_{\lambda _{\textrm{IR}}}$ is the PI of a wavelength in the infrared spectral range (850 nm or 940 nm).

Larger RoR corresponds to lower $\mathrm {SpO_2}$. Pulse oximeters apply linear or quadratic calibration models to estimate $\mathrm {SpO_2}$ based on RoR:

10 $$\begin{aligned} \mathrm {SpO_2} = A \times \textrm{RoR}^2 + B \times \textrm{RoR} + C, \end{aligned}$$

where *A*, *B*, and *C* are calibration constants determined experimentally.

For $\mathrm {SpO_2}$ estimation, we developed general population calibration models across all photon-skin simulations and all sensor configurations. For each general calibration model, we considered arterial oxygen saturation $\mathrm {SaO_2}$ ranging from 70% to 100% in 10% steps for systole and diastole. Melanin concentrations $C_\textrm{Mel}$ ranging from 2.55% to 30.5% were used to simulate Fitzpatrick Skin Types I to VI ^36^. We included discrete steps of melanin concentrations $C_\textrm{Mel}$ of 2.55%, 5.5%, 10.5%, 15.5%, 20.5%, 25.5%, and 30.5%. Wavelength combinations for pulse oximeter simulations consisted of one wavelength in the red spectrum and one in the infrared spectrum. To analyse the effect of wavelength selection in the red and infrared spectrum, we included the wavelength combinations 624 nm/850 nm, 624 nm/940 nm, 660 nm/850 nm, and 660 nm/940 nm.

MC simulations used a specific parameter seed for the random number generator (RNG) ^15^. The initial seed influenced the variability in the photon interaction sequence and was responsible for variations in MC simulation runs, including photon path length or scattering angle according to tissue properties. The parameter seed was interpreted as natural interpersonal variability (e.g., tissue layer thickness, skin temperature, or perfusion) and was used to establish a virtual study cohort for our simulations. For all parameter combinations (beam profile, beam incidence angle, wavelength, source-detector distance, arterial oxygen saturation $\mathrm {SaO_2}$, melanin concentration $C_\textrm{Mel}$, and systole and diastole), MC simulations were performed 25 times with $5\times 10^8$ photon packets per simulation.

### Evaluation

Based on the generalised population calibration model, we analysed mean absolute error (MAE) for $\mathrm {SpO_2}$ estimation to compare different sensor configurations. The MAE of the $\mathrm {SpO_2}$ estimates was computed across the set $\mathscr {I}$ of simulation instances (e.g., combinations of source wavelength, sensor geometry, $C_{\textrm{Mel}}$, $\text {SaO}_2$, seed) as

11 $$\begin{aligned} \textrm{MAE} \;=\; \frac{1}{|\mathscr {I}|}\,\sum _{i\in \mathscr {I}} \left| \,\textrm{SpO}_{2,i} \;-\; \textrm{SaO}_{2,i}\,\right| , \end{aligned}$$

where $\textrm{SpO}_{2,i}$ denotes the estimated saturation obtained from the calibration model in Eq. 10 based on $\textrm{RoR}_i$ from Eq. 9 For stratified analyses (e.g., fixed geometry or melanin level), $\mathscr {I}$ was restricted to the corresponding subset.

The Wilcoxon signed-rank test ($\alpha = 0.05$) was used to analyse differences between paired sensor configurations in photon-skin simulations. Data distribution normality was analysed using the Shapiro-Wilk test.

SNR was estimated using the AC level mean, defined as the difference between the mean PPG signal intensities during systole and diastole: $\mu _{\mathrm {AC \ level}} = I_{\textrm{dia}} - I_{\textrm{sys}}$. Noise was determined according to the shot noise of photon detection in our MC Simulations. Detected photon counts were assumed to follow Poisson statistics, their variance equals their mean, $Var[I^*] = \mathbb {E}[I]$, where $E(I^*)$ is the expected value of the detected intensity, and the corresponding standard deviation is $\sigma _I = \sqrt{\mathbb {E}[I]}$. The shot noise represents the fundamental limit of detection noise ^37^. The standard deviation $\sigma _{AC} = \sqrt{I_{\textrm{sys}} + I_{\textrm{dia}}}$ reflects the inherent statistical uncertainty of photon counting. Thus, the SNR was derived as:

12 $$\begin{aligned} \textrm{SNR} = \frac{I_{\textrm{dia}} - I_{\textrm{sys}}}{\sqrt{(I_{\textrm{dia}} + I_{\textrm{sys}})}} = \frac{\mu _{\textrm{AC}}}{\sigma _{AC}}. \end{aligned}$$

## Results

**Fig. 3:**
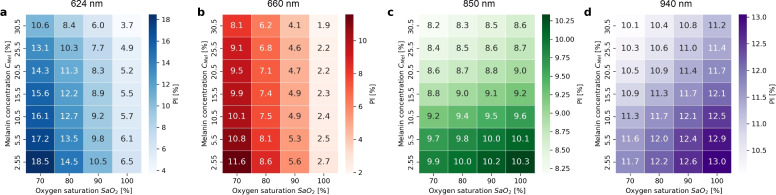
Perfusion Index (PI) for various wavelength per melanin concentration $C_\textrm{Mel}$ and arterial oxygen saturation $\mathrm {SaO_2}$ averaged across bream profiles and incidence angle (LED, VCSEL 0°, VCSEL 45°, and VCSEL − 45°). (**a**) 624 nm. (**b**) 660 nm. (**c**) 850 nm. (**d**) 940 nm.

PI decreased with increasing arterial oxygen saturation $\mathrm {SaO_2}$ and increasing melanin concentration $C_\textrm{Mel}$ for the red spectrum (see Fig. 3). In the infrared spectrum, PI decreased with decreasing arterial oxygen saturation $\mathrm {SaO_2}$ and increasing melanin concentration $C_\textrm{Mel}$. In the red spectrum, 624 nm achieved the highest PI with 18.5% (see Fig. 3a) followed by 660 nm with 11.6% (see Fig. 3b). In the infrared spectrum, 940 nm achieved a higher PI with 13.0% (see Fig. 3d) compared to 850 nm with 10.3% (see Fig. 3c). The difference between the highest and lowest PI depending on arterial oxygen saturation $\mathrm {SaO_2}$ and melanin concentration $C_\textrm{Mel}$ decreased with increasing wavelength in the red spectrum and increased in the infrared spectrum with increasing wavelength.

**Fig. 4:**
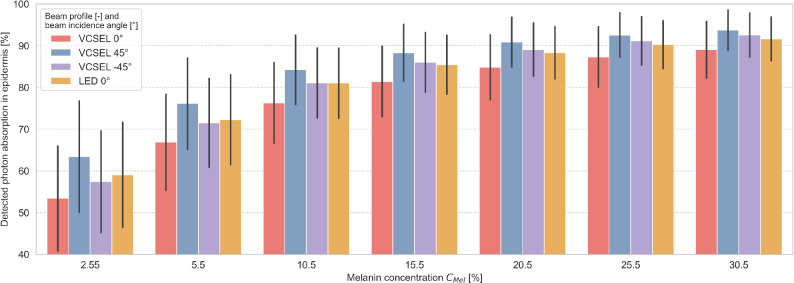
Photon absorption in the epidermis for various beam profile and beam incidence angle combinations per melanin concentration $C_\textrm{Mel}$ averaged for all wavelength (624, 660, 850, and 940 nm). Error bars indicate standard deviation due to absorption differences induced by parameter seeds, arterial oxygen saturation $\mathrm {SaO_2}$, and source-detector distance.

Figure 4 shows the absorption fractions of the total absorption of detected photons in the epidermis for different beam profiles and beam incidence angles in dependence of melanin concentration $C_\textrm{Mel}$ ranging from 2.55 to 30.5%. The detected photons emitted into tissue with VCSEL 0° showed the lowest absorption in the epidermis compared to all other combinations of beam profile and beam incidence angle, independent of melanin concentration $C_\textrm{Mel}$. The detected photons emitted into the tissue by VCSEL 45° showed the highest epidermal absorption, regardless of melanin concentration $C_\textrm{Mel}$. Absorption in the epidermis increased with increasing melanin concentration $C_\textrm{Mel}$ for all beam profiles and beam incidence angles.

**Fig. 5:**
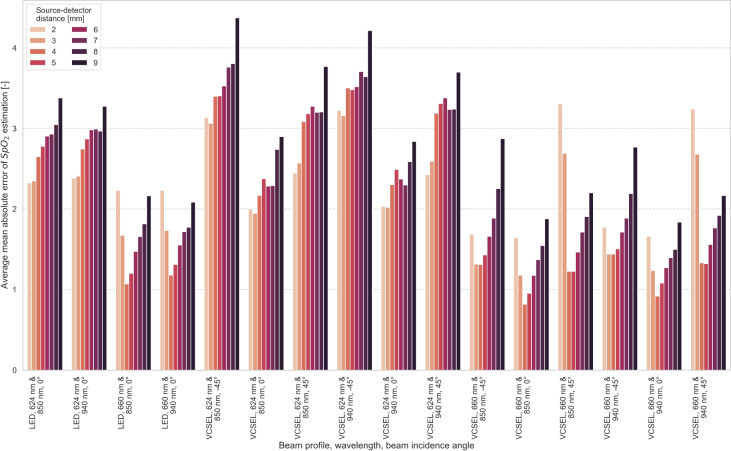
Average mean absolute error (MAE) for estimating oxygen saturation $\mathrm {SpO_2}$ vs. source-detector distance across melanin concentrations $C_\textrm{Mel}$ and arterial oxygen saturations $\mathrm {SaO_2}$ (see Eq. 11). Beam profiles include LED and VCSEL. Beam incidence angle include 0° for LED and VCSEL, 45° and -45° for VCSEL. Wavelength combinations include red (624 nm, 660 nm) and infrared (850 nm, 940 nm) spectrum.

**Fig. 6:**
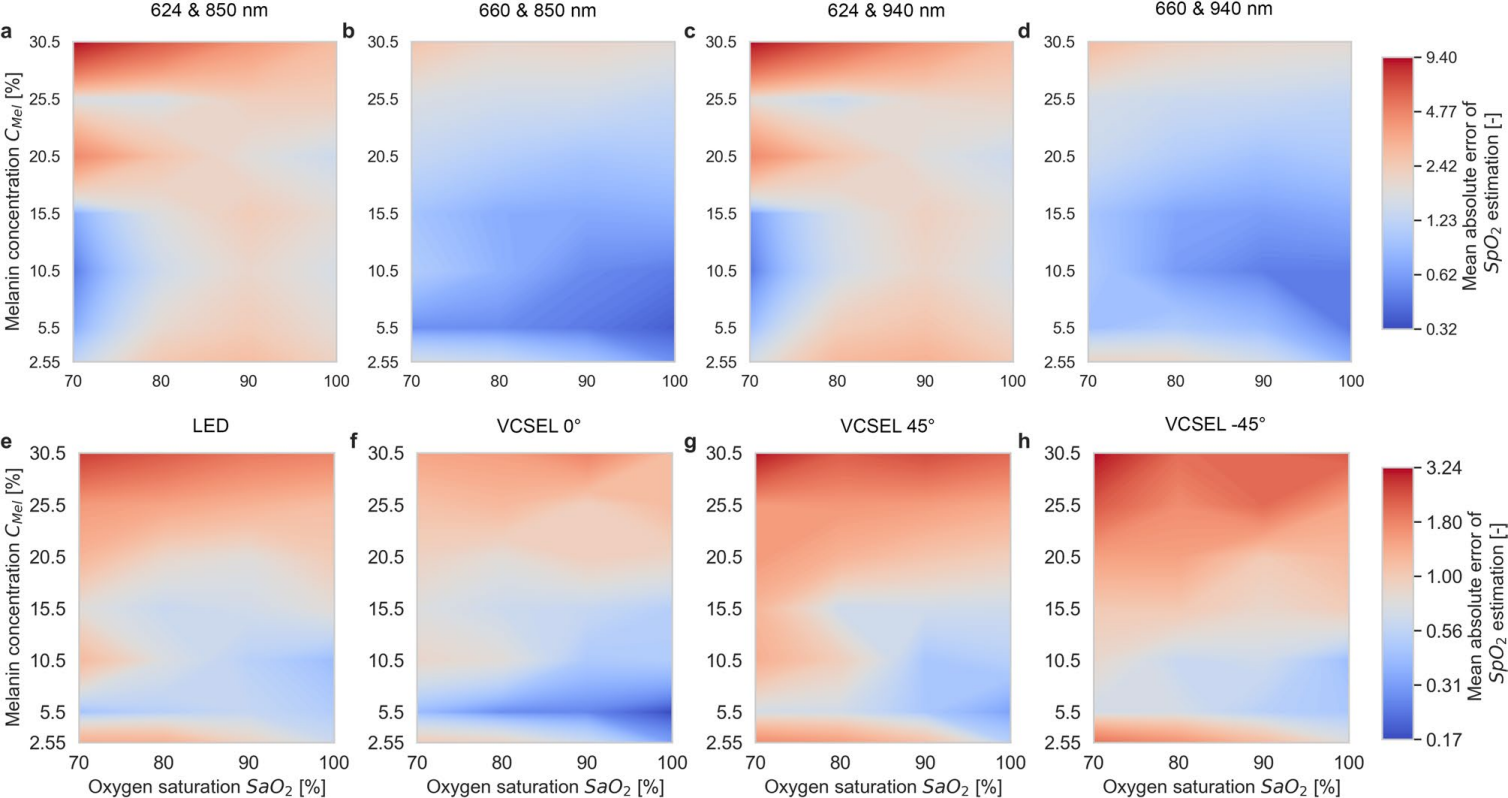
Mean absolute error (MAE) for estimating oxygen saturation $\mathrm {SpO_2}$ with melanin concentration $C_\textrm{Mel}$ and arterial oxygen saturation $\mathrm {SaO_2}$ (see Eq. 11). Subplots are based on different wavelength combinations and beam profiles including beam incidence angles. (**a**) 624 nm and 850 nm. (**b**) 660 nm and 850 nm. (**c**) 624 nm and 940 nm. (**d**) 660 nm and 940 nm. (**e**) LED. (**f**) VCSEL 0°. (**g**) VCSEL 45°. (**h**) VCSEL $\mathrm {-45}$°. Plots were generated using a kernel density estimation with 100 discrete contour levels.

Table 2 summarises the calibration constants (A,B,C) derived for $\mathrm {SpO_2}$ estimation models (see Eq. 10) across all wavelength combinations (624, 660, 850, and 940 nm). The derived calibration constants are similar to values reported in literature ^34,38,39^. We attributed deviations to dataset-specific calibrations.

**Table 2:** Comparison of quadratic $SpO_2$ calibration constants *A*, *B*, and *C* (see Eq. 10) of our calibration models (including wavelengths 624, 660, 850, and 940 nm) with literature.

Calibration model	A	B	C
Our model (624, 850 nm)	0.62	− 27.25	116.34
Our model (624, 940 nm)	2.56	− 35.60	115.98
Our model (660, 850 nm)	1.78	− 36.77	109.08
Our model (660, 940 nm)	5.59	− 46.98	108.94
Guo et al. ^38^	2.23	− 35.65	118.1
MAX30101 ^39^	1.60	− 34.66	112.69
Venema et al. ^40^	14.8	122.6	176.6

Figure 5 shows the average $\mathrm {SpO_2}$ estimation MAE per source-detector distance, beam profile, and beam incidence angle. Wavelength combinations for $\mathrm {SpO_2}$ estimation included the red spectrum (624 nm and 660 nm) and the infrared spectrum (850 nm and 940 nm). The source-detector distances with the lowest average $\mathrm {SpO_2}$ estimation MAE varied depending on the wavelength combination. For wavelength combinations including 624 nm, the optimal distance ranged between 2 mm and 3 mm and for 660 nm between 3 mm and 5 mm. The wavelengths in the red spectral range had a greater influence on the average absolute $\mathrm {SpO_2}$ estimation error with an average deviation of $\mathrm {1.3\pm 0.5}$ between 624 nm and 660 nm compared to the wavelengths in the infrared range with an average deviation of $\mathrm {0.07\pm 0.04}$. For a given source-detector distance, VCSEL 0° yielded the lowest $\mathrm {SpO_2}$ errors compared to all other beam profiles, beam incidence angles, and regardless of wavelength. At 660 nm, VCSEL $\mathrm {-45^\circ }$ performed better than VCSEL $\mathrm {45^\circ }$ for short source-detector distance (2 to 3 mm). But VCSEL $\mathrm {45^\circ }$ outperformed VCSEL $\mathrm {-45^\circ }$ for increasing source-detector distance above 3 mm. At 624 nm, VCSEL $\mathrm {45^\circ }$ outperformed VCSEL $\mathrm {-45^\circ }$ across all source-detector distances.

The $\mathrm {SpO_2}$ estimation MAE is shown in Fig. 6a to d. $\mathrm {SpO_2}$ estimates using 624 nm (see Fig. 6a, c) resulted in a higher MAE compared to 660 nm (see Fig. 6b, d). $\mathrm {SpO_2}$ estimates using 850 nm (see Fig. 6a, b) instead of 940 nm (see Fig. 6c, d) resulted in a lower absolute error, especially at decreasing melanin concentration $C_\textrm{Mel}$. The $\mathrm {SpO_2}$ estimation MAE was influenced by melanin concentration $C_\textrm{Mel}$ and arterial oxygen saturation $\mathrm {SaO_2}$. The impact of different beam profiles and beam incidence angles on the $\mathrm {SpO_2}$ estimation MAE is shown in Fig. 6e to h. VCSEL 0° (see Fig. 6f) achieved the lowest absolute errors compared to LED (see Fig. 6e), VCSEL 45° (see Fig. 6g), and VCSEL $\mathrm {-45}$° (see Fig. 6h). The $\mathrm {SpO_2}$ estimation MAE fluctuated with varying melanin concentration $C_\textrm{Mel}$ and arterial oxygen saturation $\mathrm {SaO_2}$.

**Fig. 7:**
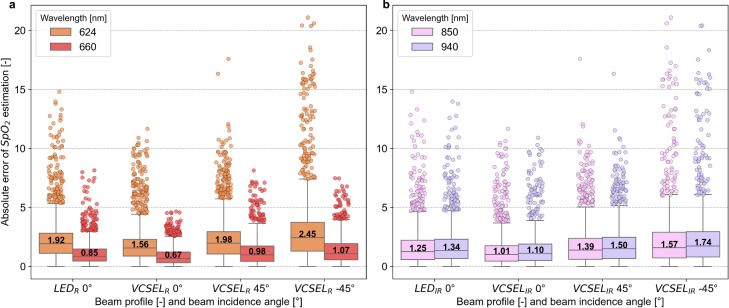
Mean absolute error (MAE) of oxygen saturation $\mathrm {SpO_2}$ estimation for beam profiles and incidence angles (LED, VCSEL 0°, VCSEL 45°, VCSEL $\mathrm {-45}$°) (see Eq. 11). Each box plot is based on more than 1500 data points. (**a**) red spectrum (624 nm, 660 nm). (**b**) infrared spectrum (850 nm, 940 nm).

The absolute error of $\mathrm {SpO_2}$ estimation is shown in Fig. 7. The median $\mathrm {SpO_2}$ estimation absolute error of wavelength combinations including 624 nm was higher for each beam profile and beam incidence angle compared to 660 nm (see Fig. 7a). The $\mathrm {SpO_2}$ estimation MAE at 850 nm was lower than that at 940 nm regardless of the beam profile and beam incidence angle (see Fig. 7b). In the red spectral range, at 660 nm, the absolute error decreased by $54.6\% \pm 2.9$ compared to 624 nm. In the infrared spectral range, light sources at 850 nm reduced the absolute error by $8.4\% \pm 1.0$ compared to 940 nm. The median of VCSEL 0° consistently achieved a lower absolute error compared to the LED. VCSEL 0° decreased the absolute error of $\mathrm {SpO_2}$ estimation by $19.3\% \pm 1.4$ compared to LED. The LED always achieved a lower absolute error than VCSEL 45° and followed by VCSEL $\mathrm {-45}$°. The lowest median absolute error of $\mathrm {SpO_2}$ estimation in the red spectrum was at 660 nm and VCSEL 0° with 0.67 and in the infrared spectrum at 850 nm and VCSEL 0° with 1.01. All differences in absolute errors of $\mathrm {SpO_2}$ estimation were statistically significant (see Table 3).

**Table 3:** Statistical test results comparing the absolute error of oxygen saturation $\mathrm {SpO_2}$ estimation between different wavelengths (624 nm and 660 nm, 850 nm and 940 nm) and source configurations.

Hypothesis	Test	*p*-value
Error $\textrm{LED}_\textrm{624 nm} > \textrm{Error LED}_\textrm{660 nm}$	Wilcoxon	$< 0.001$
Error $\textrm{ VCSEL 0 }^\circ ~_\textrm{624 nm} > \textrm{Error VCSEL 0}^\circ ~_\textrm{660 nm}$	Wilcoxon	$< 0.001$
Error $\textrm{VCSEL 45}^\circ ~_\textrm{624 nm} > \textrm{Error VCSEL 45}^\circ ~_\textrm{660 nm}$	Wilcoxon	$< 0.001$
Error $\textrm{VCSEL}~-45^\circ ~_\textrm{624 nm} > \textrm{Error}\, \textrm{VCSEL}\, {-45}^\circ ~_\textrm{660 nm}$	Wilcoxon	$< 0.001$
Error $\textrm{LED}_\textrm{940 nm} > \textrm{Error LED}_\textrm{850 nm}$	Wilcoxon	$1.7\times 10^{-03}$
Error $\textrm{VCSEL 0}^\circ ~_\textrm{940 nm} > \textrm{Error VCSEL 0}^\circ ~_\textrm{850 nm}$	Wilcoxon	$< 0.001$
Error $\textrm{VCSEL 45}^\circ ~_\textrm{940 nm} > \textrm{Error VCSEL 45}^\circ ~_\textrm{850 nm}$	Wilcoxon	$< 0.001$
Error $\mathrm {VCSEL -45}^\circ ~_\textrm{940 nm} > \textrm{Error}\,\textrm{VCSEL}\,{-45}^\circ ~_\textrm{850 nm}$	Wilcoxon	$< 0.001$

The Wilcoxon signed-rank test was used due to non-normal data distributions. *P*-values are presented in scientific notation, with values below 0.05 indicating statistically significant differences.

**Fig. 8:**
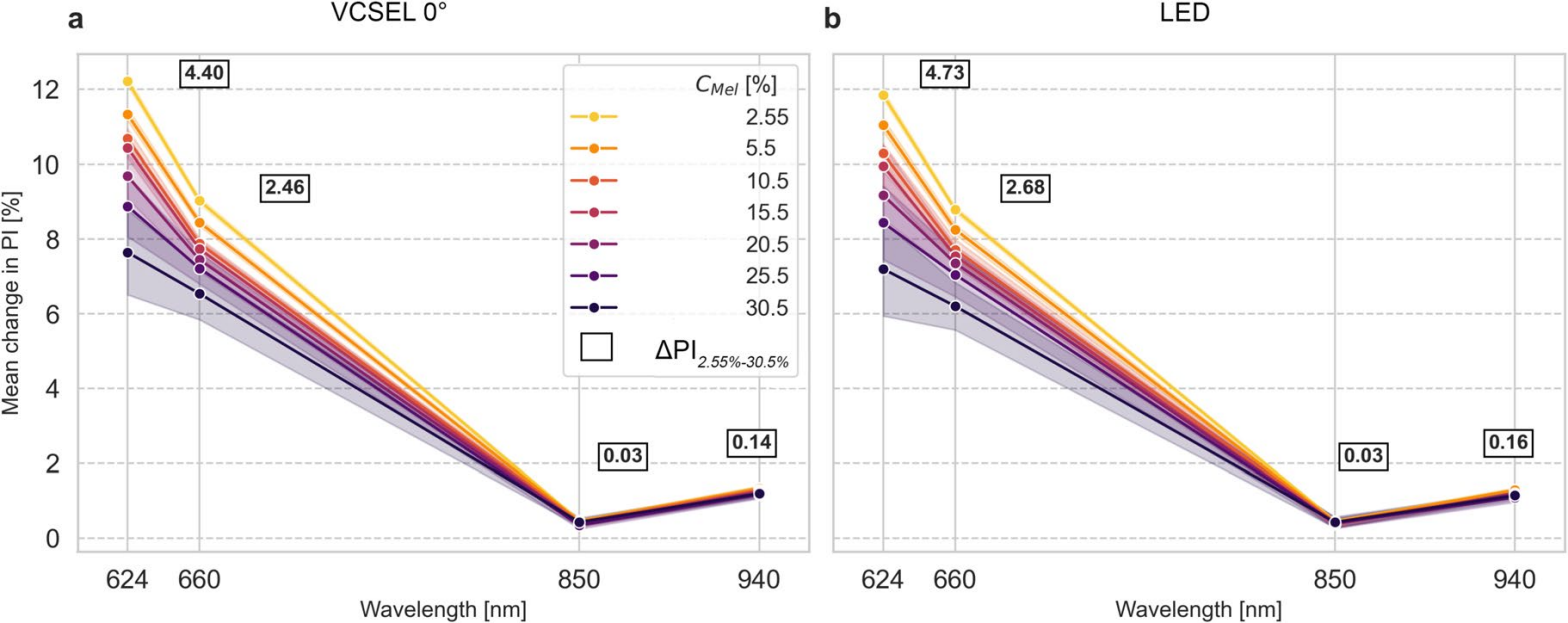
Mean change of perfusion index (PI) from 70% to 100% arterial oxygen saturation $\mathrm {SaO_2}$ for wavelengths 624 nm, 660 nm, 850 nm, and 940 nm. Melanin concentration $C_\textrm{Mel}$ is ranging from 2.55 to 30.5%. The annotated values highlight the difference in mean change of PI between melanin concentrations C_Mel_ of 2.55% and 30.5%. Line plots are used to illustrate the relationship of wavelengths. No continuum can be assumed between analysed wavelengths. a: Mean change of PI for beam profiles $\textrm{VCSEL}_\textrm{R}$ and $\textrm{VCSEL}_\textrm{IR}$ with a beam incidence of 0°. b: Mean change of PI for beam profiles $\textrm{LED}_\textrm{R}$ and $\textrm{LED}_\textrm{IR}$.

Figure 8 shows the change in PI resulting from the variation in arterial oxygen saturation $\mathrm {SaO_2}$ from 70 to 100%. The mean change of PI for the beam profile and incidence beam angle VCSEL 0° is shown in Fig. 8a and for LED in Fig. 8b. The difference in the mean change of PI between melanin concentrations C_Mel_ of 2.55% and 30.5% decreased for the red spectrum from 4.40 percentage points at 624 nm to 2.46 percentage points at 660 nm for VCSEL 0° and from 4.73 percentage points at 624 nm to 2.68 percentage points at 660 nm for LED. The mean change of PI between melanin concentrations C_Mel_ of 2.55% and 30.5% increased for the infrared spectrum from 0.03 percentage points at 850 nm to 0.14 percentage points at 940 nm for VCSEL 0° and from 0.03 percentage points at 850 nm to 0.16 percentage points at 940 nm for LED. The mean change in PI of VCSEL 0° was always higher for LED except at 850 nm.

**Fig. 9:**
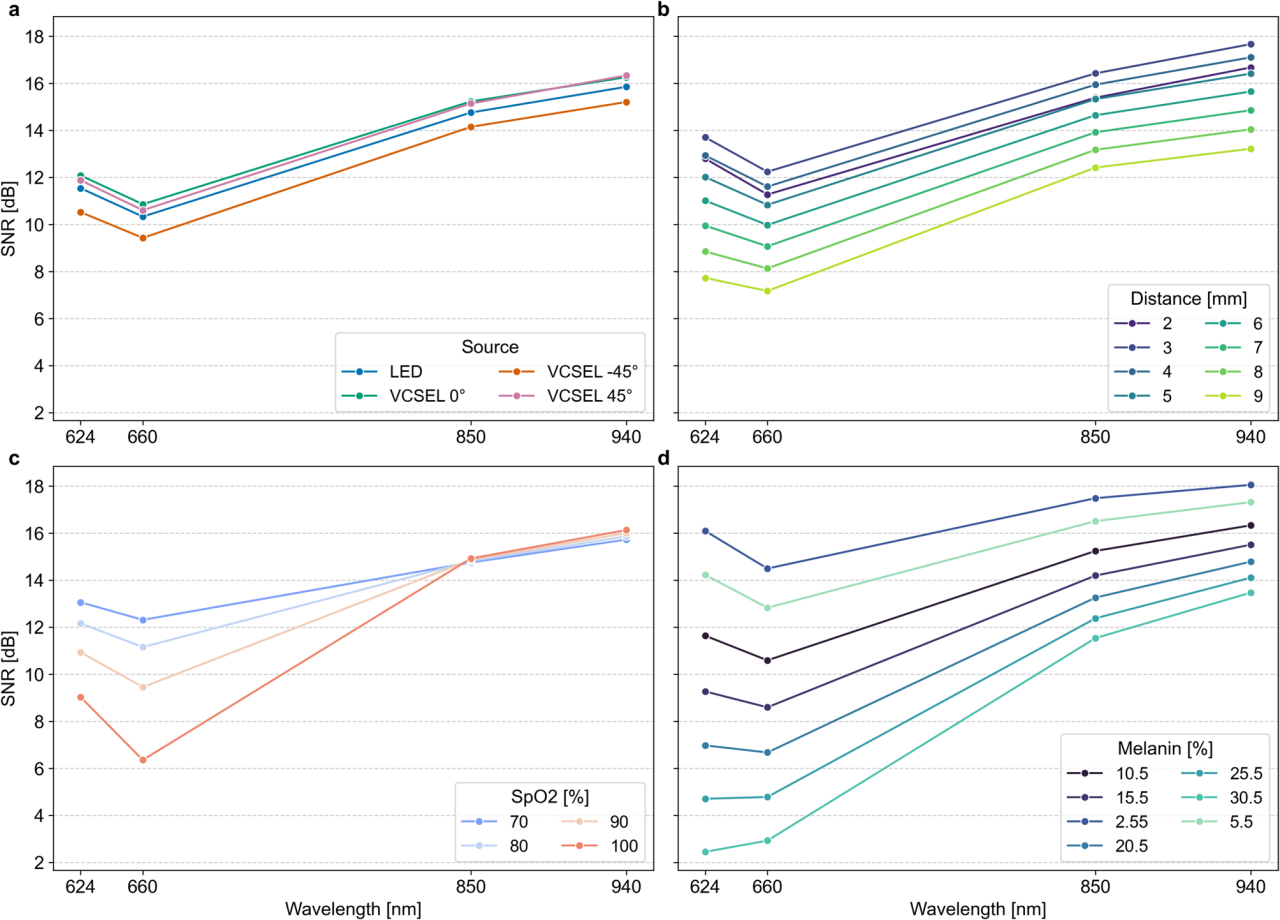
Signal to noise ratio (SNR) for wavelengths 624 nm, 660 nm, 850 nm, and 940 nm. Line plots are used to illustrate the relationship of wavelengths. No continuum can be assumed between analysed wavelengths. (**a**) SNR depending on beam profile and angle (LED, VCSEL 0°, VCSEL − 45°, and VCSEL 45°). (**b**) SNR depending on source-detector distances ranging from 2 to 9 mm. (**c**) SNR depending on $SaO_2$ saturation ranging from 70 to 100%. (**d**) SNR depending on melanin concentration ranging from 2.55 to 30.5%.

With increasing wavelength, SNR decreased within the red spectrum and increased within the infrared spectrum (see Fig. 9a). Among beam profiles and beam incidence angles, VCSEL 0° yielded the highest SNR for all wavelengths except at 940 nm. SNR of the LED beam profile was consistently lower compared to VCSEL 0°. VCSEL $\mathrm {-45}$° showed the lowest SNR across beam profiles and beam incidence angles. SNR increased with source–detector distance from 2 to 3 mm, followed by a subsequent decline from 3 to 9 mm (see Fig. 9b). Figure 9c shows the dependence of the SNR on wavelength and $\mathrm {SaO_2}$ concentration. In the red spectral range, SNR decreased with increasing $\mathrm {SaO_2}$ concentration, whereas in the infrared range SNR increased. Elevated melanin concentration reduced SNR for all wavelengths (see Fig. 9d). Below 30.5% melanin concentration, SNR at 624 nm exceeded that at 660 nm, except at 30.5%. With increasing melanin concentration, the relative advantage of SNR at 624 nm compared to 660 nm decreased until SNR at 660 nm surpassed that at 624, nm.

## Discussion

Wearable PPG devices are used for vital parameter estimation, including heart beat and oxygen saturation $\mathrm {SpO_2}$. For the first time, we analysed the essential parameter space for reflective PPG sensor design (i.e., wavelength, beam profile, beam incidence angle, and source-detector distance) with the objective to maximise PI and minimise $\mathrm {SpO_2}$ estimation error across skin types (i.e., melanin concentration).

We explored typical wavelengths in the red spectrum (624 nm and 660 nm) and in the infrared spectrum (850 nm and 940 nm). Moço et al. ^12^ analysed PI for wavelengths from 450 to 1000 nm, but did not consider absorption due to melanin concentration nor how to maximise PI. However, melanin concentration has a critical effect on PPG-based health monitoring, where darker skin types often suffer from larger error^6,9^. Our skin-photon model represents melanin-related absorption to explore how PPG sensor design can be optimised for vital parameter estimation (i.e., PI and $\mathrm {SpO_2}$) depending on skin type. Our results for PI across melanin concentrations $C_\textrm{Mel}$ ranging from 2.55 to 30.5% are in line with literature ^12,14^. Moreover, our findings highlight that with increasing signal quality (i.e., PI) the $\mathrm {SpO_2}$ estimation error may increase too.

In our MC simulations, we used shot noise to represent the fundamental lower bound of measurement uncertainty. In particular, we avoid volatile noise effects of the detector and electronics. For example, photodetectors register photon events as discrete and independent events and the resulting statistics follow a Poisson distribution. When photon events increase, the SNR improves with the square root of the detected photon number. With the simulated shot noise, we focus on the basic source of noise and thus obtain an estimate of optimal SNR. Further noise sources (including dark current noise and amplifier noise) might add noise contributions in practical implementations. Our approach allowed us to compare and interpret different sensor configurations without distortions due to device-specific and manufacturer-specific characteristics.

### Light wavelength effect

We showed that the selected wavelength influenced PI magnitude (see Fig. 3). PI increased with increasing difference between absorptions of $\textrm{O}_{2}\textrm{Hb}$ and $\textrm{HHb}$. When wavelength was reduced within the red spectrum, PI and the difference between absorptions of $\textrm{O}_{2}\textrm{Hb}$ and $\textrm{HHb}$ increased. Conversely, when wavelength was decreased within the infrared spectrum, the absorption difference declined, which corresponds to theoretical analyses ^28^. In summary, both, decreasing wavelength in the red spectrum and increasing wavelength in the infrared range, can maximise PI.

We found that wavelength influenced the relationship of PI and melanin concentration C_Mel_ (see Fig. 3). With decreasing wavelength, melanin-related absorption in the epidermis increased and consequently PI decreased. To optimise sensor design for PI, wavelength shall be selected to maximise PI, based on (1) maximising the difference between absorptions of $\textrm{O}_{2}\textrm{Hb}$ and $\textrm{HHb}$ and (2) minimising absorption due to melanin. By maximising PI, the accuracy of vital parameter estimation methods that rely on maximising PPG amplitude (e.g., heart beat estimation) will improve.

We showed that the selected wavelength influenced $\mathrm {SpO_2}$ estimation (see Fig. 7). $\mathrm {SpO_2}$ estimates were determined using a calibration curve based on RoR values, thus depended on PI (see Eq. 9). Melanin-related absorption affects PI more prominently in the red spectrum as opposed to the infrared spectrum. When melanin concentrations $C_\textrm{Mel}$ in the epidermis increase, RoR variation from the calibration curve increases, leading to increased $\mathrm {SpO_2}$ estimation error (see Fig. 8). With increasing difference in absorption between $\textrm{O}_{2}\textrm{Hb}$ and $\textrm{HHb}$, the effect of melanin-related absorption increased too and consequently $\mathrm {SpO_2}$ estimation errors increased. To optimise sensor design for $\mathrm {SpO_2}$ estimation, wavelengths should be selected to (1) minimise the difference in absorption between $\textrm{O}_{2}\textrm{Hb}$ and $\textrm{HHb}$ and (2) minimise the influence of melanin-related absorption.

Absolute $\mathrm {SpO_2}$ estimation error increased with melanin concentration $C_\textrm{Mel}$, peaking at $C_\textrm{Mel}=30.5\%$, for all combinations of wavelengths, beam profiles, and beam incidence angles (see Fig. 6), which is in line with literature ^3^. The result provides further support to our previous hypothesis that calibration curves adapted to specific melanin concentration ranges may reduce $\mathrm {SpO_2}$ estimation error ^13^.

We observed that $\mathrm {SpO_2}$ estimation error was affected by melanin concentration $C_{\textrm{Mel}}$ and arterial oxygen saturation $\mathrm {SaO_2}$ settings (see Fig. 6). Different wavelengths, beam profiles, and beam incidence angles (see Fig. 4 and Fig. 7) lead to similar MAE scatter. The differences in the $\mathrm {SpO_2}$ estimation error between 624 nm and 660 nm as well as between 850 nm and 940 nm were significant, regardless of the beam profile and beam incidence angle.

Wavelength choice had a direct influence on SNR. As PI increased in the red and infrared spectrum, SNR increased too. However, wavelengths that maximise PI and SNR increase $\mathrm {SpO_2}$ estimation error. Thus, for $\mathrm {SpO_2}$ estimation, we recommend higher wavelengths in the red spectrum (e.g., 660 nm) and lower wavelengths in the infrared spectrum (e.g., 850 nm). To maximise PI, an opposite wavelength choice is recommended: a lower wavelength in the red spectrum (e.g., 624 nm) combined with a higher wavelength in the infrared spectrum (e.g., 940 nm).

In addition to wavelength-dependent absorption coefficients $\mu _a$, the effective absorption in skin tissue is influenced by the mean photon pathlength, which is also wavelength-dependent ^41^. Variations in photon pathlength modulate the amount of absorbed light and thereby affect PI and $\mathrm {SpO_2}$ estimation.

### Light source effect

We included VCSELs in our light source analysis due to their characteristic beam incidence angles and non-Lambertian beam profile. We showed that (1) increasing photon path through tissue with blood volume, and (2) decreasing absorption caused by tissue without blood volume, can increase PI. Detected photons travel through tissue in a banana-shaped curve ^10^, where the curve can be controlled by the emission angle of the light source ^11^. In our present analysis, photons emitted perpendicular to the skin surface (i.e., at 0°) had the shortest path through tissue without blood volume (e.g., epidermis), and thus increased PI. Photons emitted with positive orientation angle (towards the detector) mainly penetrated skin layers close to the skin surface. For VCSEL 45°, the photon path in tissue with blood volume decreased and in tissue without blood volume increased, compared to VCSEL 0° and therefore PI decreased.

A light source with negative orientation angle (emitting photons in opposite direction of the detector) increased photon path in tissue with and without blood volume. We show that for VCSEL $\mathrm {-45}$°, PI increased. Hence, the effect of the extended photon path in tissue with blood volume dominated over the effect of the extended photon path in tissue without blood volume. Consequently, in wearable devices VCSEL $\mathrm {-45}$° can minimise device space needs (i.e., source-detector distance) and maximise PI. However, our results also showed that VCSEL $\mathrm {-45}$° increased $\mathrm {SpO_2}$ estimation error. In addition, longer photon paths through tissue could elevate the effect of motion artefacts on the PPG signal ^42^. If motion artefacts are expected, e.g. due to the wearable device attachment or body position, VCSEL 45° may still provide better performance for vital sign estimation compared to $\mathrm {-45}$°.

LED beam profiles showed larger relative light intensity across a wider range of radiation angles compared to the VCSEL profiles (see Fig. 2). Consequently, LEDs had a larger ratio of positive and negative photon incidences than VCSELs, when oriented perpendicular (0°) at the skin surface. Still, the ratio of photons with positive incidence paths outweighed those with negative incidences, resulting in lower PI compared to VCSEL 0°.

Moreover, VCSEL 0° reduced $\mathrm {SpO_2}$ estimation error compared to LEDs at the same incidence angle by $19.3\% \pm 1.4$ (see Fig. 7). Detected photons for VCSEL 0° passed through the epidermis with a shorter photon path compared to all other configurations of VCSEL, LED, and incidence angles. VCSEL 0° reduced the absorption of detected photons by an average of $\mathrm {5.1\pm 1.3\%}$ relative to LED (see Fig. 4). Thus, the influence of melanin-related absorption was reduced for VCSEL 0° compared to all other configurations of VCSEL, LED, and incidence angles, resulting in minimal $\mathrm {SpO_2}$ estimation error.

VCSEL 0° consistently yielded the lowest $\mathrm {SpO_2}$ errors across all source-detector distances, thus confirms its advantage over LEDs. VCSEL $\mathrm {45^\circ }$ consistently exhibited lower MAE than VCSEL $\mathrm {-45^\circ }$, regardless of source-detector distances. However, the performance of VCSEL $\mathrm {45^\circ }$ and VCSEL $\mathrm {-45^\circ }$ depended on both wavelength and source-detector distance. For VCSEL $\mathrm {45^\circ }$ and VCSEL $\mathrm {-45^\circ }$, beam profile should be aligned with source–detector distance to achieve minimal $\mathrm {SpO_2}$ error, whereas VCSEL 0° remains the best performing light source configuration overall.

In summary, VCSELs provide an advantage for wearable PPG systems compared to LED-based beam profiles for heart beat and $\mathrm {SpO_2}$ estimation. VCSEL 0° showed the best SNR with an average of $\mathrm {12.1\pm 4.8 dB}$ across all wavelengths and thus represents an optimal light incidence angle, without considering other constraints, e.g. space limitations. We derived SNR from the AC component (i.e., PI) and from the simulated light intensities $I_{\textrm{sys}}$ and $I_{\textrm{dia}}$. When beam incidence angle decreased, PI increased and noise decreased for all wavelengths, due to the prolonged photon path in tissue with blood volume.

### Source-detector distance

Our results suggest that the optimal source-detector distance for $\mathrm {SpO_2}$ estimation is wavelength specific. In the red spectrum, a change in wavelength leads to a larger change in optimal source-detector distance than in the infrared spectrum (see Fig. 5). Moreover, the optimal source-detector distance for $\mathrm {SpO_2}$ estimation within the red and infrared spectra depends on PI (see Eq. 9). Three primary parameters influence the relation of source-detector distance and PI:

1. Penetration depth increases with wavelength ^41^, thus lead to an increase of PI. Moreover, penetration depth increases with source-detector distance until a wavelength-dependent saturation point, beyond which penetration depth subsequently decreases.
2. Photon path decreases with source-detector distance ^11^, and thus reduces PI.
3. Melanin-related absorption increases as wavelength is reduced. Optimal source-detector distance decreases with increasing melanin concentration. The effect is more prominent within the red spectrum (see Fig. 3).

We observed that the tipping point of the optimal source-detector distance at 660 nm is between 4 mm to 5 mm. At 624 nm, the tipping point is at 2 mm to 3 mm.

### Summary

Across all sensor configurations, our results indicate that wavelength and source-detector geometry interact in complex ways with melanin concentration to shape PI, SNR, and $\mathrm {SpO_2}$ estimation error. The following primary insights should be considered:

1. Melanin-related absorption that influences PI, increases as wavelength decreases. The effect is more prominent within the red spectrum (see Fig. 3).
2. Within the red spectrum, a higher difference in absorption between $\textrm{O}_{2}\textrm{Hb}$ and $\textrm{HHb}$ leads to a higher difference in PI between 70% and 100% $\mathrm {SaO_2}$ (see Fig. 3).
3. The mean change in PI increases (i.e., increase of baseline PI) when (A) wavelength within the red spectrum is decreases, (B) wavelength within infrared spectrum increases, (C) with melanin-related absorption (see Fig. 8).

Two counteracting effects are evident in the red spectrum: According to Item 2 above, PI increases due to greater differences in absorption of $\textrm{O}_{2}\textrm{Hb}$ and $\textrm{HHb}$. However, according to Item 1 above, PI decreases with increasing melanin absorption. Item 2 dominates at 660 nm, where photons require a longer photon path, compared to 624 nm.

Our results highlight that design optimisation for reflective PPG sensors is multidimensional. Source wavelength, source-detector geometry may optimise PI and SNR but not $\mathrm {SpO_2}$ estimation error at the same time. Therefore, PPG sensor designs for specific applications should be based on parameter combinations that align with the primary measurement goal (e.g., robust heart rate detection, accurate $\mathrm {SpO_2}$ estimation, or balanced performance across melanin concentrations).

### Limitations

Our analysis was based on MC simulations. MC simulations approximate the real-world parameter space. Our simulation approach has been previously validated with actual measurements ^11,35^. MC simulations enable us to analyse photon-tissue interactions and explore fundamental principles of the PPG signal measurement. In contrast, real-world measurements require a sufficient study cohort that covers the entire range of melanin concentrations C_Mel_. Moreover, real-world measurements are influenced by various external and internal factors, including body temperature, sensor positioning, motion artefacts, and interpersonal variability. Consequently, a measurement-based analysis would require a sufficiently large cohort to account for variations. Based on the insights of this work, we believe that further investigations are warranted to amend empirical observations across the parameter space of reflective PPG sensor design.

The present study examines combinations of two red (624 nm, 660 nm) and two infrared (850 nm, 940 nm) wavelengths commonly deployed in PPG. The selected wavelengths sample representative design points and expose the opposing tendencies quantified herein: comparably higher PI and SNR at 624 nm and 940 nm versus lower $\mathrm {SpO_2}$ estimation error at 660 nm and 850 nm. For the selection of wavelengths in this work, a specific and tractable parameter space was chosen to investigate effects related to geometry, melanin, and $\mathrm {SaO_2}$ conditions. Future work may build on our approach to characterise trade-offs and robustness of more nuanced wavelength combinations in relation to other pulse oximeter system design choices.

Measured by the full-width-at-half-maximum (FWHM) approach, LEDs typically exhibit a spectral bandwidth of 20 nm for $\textrm{LED}_\textrm{R}$ and 42 nm for $\textrm{LED}_\textrm{IR}$ FWHM. VCSELs however, typically emit in a narrower spectral range of 4 nm for $\textrm{VCSEL}_\textrm{R}$ and 2 nm for $\textrm{VCSEL}_\textrm{IR}$ FWHM. Previous pulse oximetry studies have shown that broad LED bandwidths lead to spectral distortion due to melanin variations, thereby increasing $\textrm{SpO}_{2}$ estimation error ^26^. In contrast, the comparably narrow spectra of VCSELs are largely unaffected. Our MC simulations provide new insight on how light sources affect $\mathrm {SpO_2}$ estimation error. In particular, our analysis focussed on sensor configuration (beam profile, incidence angle, and source-detector distance): (1) We separate bandwidth-related error from geometry-related contributions, and (2) highlight that source-detector geometry itself has a measurable effect on $\mathrm {SpO_2}$ error and SNR, independent of spectral distortion. In practice, both bandwidth and geometry affect $\mathrm {SpO_2}$ estimation. Nevertheless, our results show that even if bandwidth limitations are acknowledged, a specific choice of source geometry (e.g., VCSEL 0°) can reduce $\mathrm {SpO_2}$ estimation error by $19.3\% \pm 1.4$ compared to LEDs (see Fig. 7). Thus, while bandwidth has been established in literature as a critical metric, our results show the relevance of source-detector geometry optimisation as a complementary design aspect for pulse oximeters. In conclusion, both spectral and geometrical design must be considered, and future work should address their combined influence on $\mathrm {SpO_2}$ estimation.

Future work should include explicit analysis of wavelength-dependent photon pathlength. Considering both the absorption coefficients and the optical pathlength in tissue could further improve the interpretation of $\mathrm {SpO_2}$ estimation errors.

## Conclusion

We investigated the influence of melanin concentration C_Mel_ and reflective PPG sensor design (including wavelength, beam profiles, and beam incidence angle) on PI, signal quality, $\mathrm {SpO_2}$ estimation, and SNR. Our results provide a comprehensive overview on the opportunities for PPG sensor design with regard to essential design parameters.

In the red spectrum, PI and SNR decreased and $\mathrm {SpO_2}$ estimation performance improved with increasing wavelength. In the infrared spectral range, PI, SNR, and the absolute error for $\mathrm {SpO_2}$ estimation increased with increasing wavelength. A VCSEL beam profile at 0° outperformed all other beam profiles and beam incidence angles for $\mathrm {SpO_2}$ estimation and achieved the highest SNR for most wavelengths, except at 940 nm. VCSEL beam profile at -45° showed the highest PI with an average of $\mathrm {9.2\pm 4.7\%}$, but the lowest SNR too with an average of $\mathrm {10.5\pm 5.4 dB}$. Overall, our results indicated that the VCSEL beam profile at 0° is the optimal light source for a wide range of applications, as it offers a balance between high PI with an average of $\mathrm {8.7\pm 4.6\%}$, low absolute error for $\mathrm {SpO_2}$ estimation with an average of $\mathrm {1.1\pm 0.3}$, and high SNR with an average of $\mathrm {12.1\pm 4.8 dB}$.

Universal recommendations for optimal source–detector distances are not possible, as the optimal spacing depends on several aspects of the source-detector geometry, wavelength configuration, and measurement site. Instead, optimal distances should be determined through simulation or empirical testing.

The optimum source wavelength depended on the application: For heart rate measurement, where PI should be maximised, 624 nm in the red spectrum and 940 nm in the infrared spectrum were optimal choices. For $\mathrm {SpO_2}$ estimation, which is affected by variations in melanin concentration, sensor systems designed to maintain a constant change in RoR with respect to melanin concentration $C_\textrm{Mel}$ are recommended. Wavelengths of 660 nm in the red spectral range, 850 nm in the infrared spectrum, and VCSEL 0° minimised $\mathrm {SpO_2}$ estimation error. The described sensor configurations for heart rate measurement and $\mathrm {SpO_2}$ estimation could enhance performance in clinical practice, especially for individuals with elevated melanin concentration C_Mel_ towards 30.5%.

## Data Availability

The datasets generated and/or analysed during the current study are available from the corresponding author on reasonable request.
